# Real-Time Ellipsometric Surface Plasmon Resonance Sensor Using Polarization Camera May Provide the Ultimate Detection Limit

**DOI:** 10.3390/bios13020173

**Published:** 2023-01-22

**Authors:** Nipun Vashistha, Marwan J. Abuleil, Anand M. Shrivastav, Aabha Bajaj, Ibrahim Abdulhalim

**Affiliations:** Department of Electro-Optic Engineering, Ilse-Katz Institute for Nanoscale Science and Technology, Ben Gurion University, Beer Sheva 84105, Israel

**Keywords:** surface plasmons, ellipsometry, polarization camera, phase measurement

## Abstract

Ellipsometric Surface Plasmon Resonance (SPR) sensors are known for their relatively simple optical configuration compared to interferometric and optical heterodyne phase interrogation techniques. However, most of the previously explored ellipsometric SPR sensors based on intensity measurements are limited by their real-time applications because phase or polarization shifts are conducted serially. Here we present an ellipsometric SPR sensor based on a Kretschmann–Raether (KR) diverging beam configuration and a pixelated microgrid polarization camera. The proposed methodology has the advantage of real-time and higher precision sensing applications. The short-term stability of the measurement using the ellipsometric parameters tanψ and cos(Δ) is found to be superior over direct SPR or intensity measurements, particularly with fluctuating sources such as laser diodes. Refractive index and dynamic change measurements in real-time are presented together with Bovine Serum Albumin (BSA)–anti-BSA antibody binding to demonstrate the potential of the developed sensor for biological sensing applications with a resolution of sub-nM and down to pM with additional optimization. The analysis shows that this approach may provide the ultimate detection limit for SPR sensors.

## 1. Introduction

Surface Plasmon Resonance (SPR) is a phenomenon of surface charge density oscillations at the metal–dielectric interface originating from the interaction of light photons with electron plasma at the metal surface. Under certain conditions of wavelength, polarization, and incidence angle, the momentum carried by the photons along the interface is transferred to the collective oscillation of free electrons as a surface electromagnetic wave, called surface plasmons [1]. In localized SPR, the excitation is on the surface of metal nanoparticles due to scattering without dependence on the polarization if the particle is spherical [2]. In the extended or propagating SPR, the polarization must be transverse magnetic (TM) because it contains an electric field component normal to the metal surface required to generate the surface charge. In this way, at resonance, the incident light energy is coupled into the surface plasmon mode field, which results in a dip in the reflectivity of light. The SPR is highly sensitive to the refractive index of the material in the vicinity of the metal surface within the range of the penetration depth of the surface plasmon field. An extremely small variation in the refractive index results in a change in information parameters in resonance wavelength, resonance angle, phase, or intensity [3].

Over the past few decades, SPR has been widely used as an optical sensing technique that can perform real-time, label-free, and high-sensitivity monitoring of molecular interactions [4,5,6,7,8]. The most widely used configuration in SPR sensing is the one based on prism coupling, called the Kretschmann–Raether (KR) configuration [1,2]. SPR biosensors can be divided according to their operating principles into angle, wavelength, intensity, or phase-interrogated devices [9,10,11,12]. The intensity and the phase of the reflected TM-polarized light both change when there is a change in the refractive index of the analyte in the vicinity of the metal film. Intensity monitoring offers the advantage of simple optical configuration requirements and has been utilized in many commercial devices [13,14]. With their complex optical configurations, phase-interrogated SPR sensors generally provide higher sensitivity and throughput and have thus emerged as prominent biosensing devices [15,16]. The sensitivity and limit of detection (LOD) of the phase SPR and intensity SPR were extensively investigated and compared in the past. The phase SPR is found to give a much stronger response to the changes in the refractive index because of the maximal change in the phase occurring at the edge of the dip of the SPR curve [17,18,19]. While light intensity measurement is a straightforward process, high-frequency light oscillation cannot be observed directly. Phase measurement is more complicated than intensity and requires more know-how in optical configuration, modulation instrument, and processing [20,21,22].

Over the past few decades, a variety of SPR-phase interrogation techniques have been developed. The major ones include interferometry, optical heterodyne, and ellipsometry-based techniques. Light modulators such as electro-optic modulators, acousto-optic modulators, photo-elastic modulators, piezo-electric transducers, and liquid crystal modulators were used in a variety of phase interrogation systems. The piezo-electric transducers were used in various interferometric phase SPR systems based on Mach–Zehnder and Michelson interferometers [23,24]. An interferometric imaging system based on spatial phase modulation was developed.

In this approach, sensing is performed by measuring the interference fringe phase shift [25]. A different approach based on optical heterodyne used acousto-optic and electro-optic modulators to produce two beams with slightly different frequencies, which interfere and result in beat frequency which is used to measure the phase difference using a phase meter [26,27,28]. The complex optical configurations of interferometric and optical heterodyne were further simplified in the single-beam interferometric techniques using a photo-elastic modulator. In these systems, the reflected beam was passed through a photo-elastic modulator which sinusoidally modulates the p component of light. The modulated signal is then used to extract the phase information [29,30]. Liquid crystal modulator-based phase SPR systems have also been developed. The phase shift between the orthogonal components of light can be accurately measured by using a phase extraction algorithm. A three-point polarimetric approach for monitoring phase information SPR using a variable liquid crystal modulator was also developed both in the angular and the spectroscopic modes [31,32].

A more simplified approach to measuring the phase shift in phase SPR sensors involves a rotating analyzer. In this approach, ellipsometry is used to extract the phase information of light. However, these approaches are limited by their mechanical rotation speed, which can also add noise to the system and is not suitable for real-time sensing applications [33,34,35]. In addition to these sensors, SPR systems combining two interrogations, spectral and phase, were also developed [36,37]. With the proposition to perform the phase change measurements in real-time, phase SPR systems with three separate cameras or a single camera with a polarization mask were mentioned originally in some patents [38,39]. In our group, a mechanically stable spectroscopic polarimetric SPR setup with no moving parts was developed using a liquid crystal tunable Achromatic wave plate (LC AchWP) of Abuleil–Abdulhalim to extract spectral ellipsometric signatures of light [40]. Our group has been developing parallel interference microscopy, vibrometry, and ellipsometry systems for the last decade using either three different cameras or spectrometers [41,42,43,44]; however, this approach makes the system bulky and expensive. In a recent work [45] on ellipsometric phase SPR systems, an ellipsometric phase SPR system was developed using a phase camera; however, in this work, they used an extra component, a quarter waveplate, to utilize the four signals from the four polarized pixels. In addition, in [45], they did not use a spectrum of spatial frequencies, meaning they needed to carefully adjust the incident beam for each analyte medium with different refractive indices. In this paper, we propose a polarimetric SPR sensor using the diverging beam approach, three points measurement using a pixelated micro-grid polarization camera, and no additional waveplates involved. The proposed method is suitable for real-time sensing applications and is only limited by the frame rate of the polarization camera. Furthermore, we investigated the stability of information parameters SPR dip, tanψ, and cosΔ in different light source environments. The stability of the aforementioned information parameters is essential for accurate dynamic measurements.

## 2. Materials and Methods

### 2.1. Phase Extraction Algorithm

The diverging beam approach in Kretschmann–Raether (KR) configuration polarimetric was described in several publications [17,32]. The approach is characterized by the cylindrical divergence of the light beam so that a range of spatial frequencies are projected towards the sample and analyzed simultaneously. We extract the ellipso-polarimetric parameters tanψ and cos(Δ) of the reflected light using a three-point polarimetric approach [33,46]. By fixing the orientation of the input polarizer, the light reflected from the sample is analyzed using three out of the four different orientations of analyzers, 0°, 45°, 90°, and 135°, forming one pixel of the microgrid polarization camera. The orientation of the input polarizer is fixed at a 45° angle with respect to the TM polarization direction so that the incident linearly polarized light contains equal amounts of TE and TM intensities which are equal to $I_{input}$. For different analytes, four intensity images $I_{0}$, $I_{45}$, $I_{90}$, and $I_{135}$, are obtained simultaneously corresponding to the four different orientations 0°, 45°, 90°, and 135° of pixelated microgrid polarization camera. The ellipso-polarimetric parameters tanψ (defined as the absolute value of the ratio between the TM to TE amplitudes, also related to the azimuth of the polarization ellipse) and cos(Δ) (Δ is the phase difference between the TM and TE components of reflected light) which are related to each other by a single complex number χ. The parameter χ is defined as the ratio of the reflected amplitudes of TM and TE polarization components,

(1) $$\chi =\left|\frac{r_{TM}}{r_{TE}}\right|exp^{i\left(\phi_{TM}-\phi_{TE}\right)}$$

here, $\tan\left(\psi \right)=\left|\frac{r_{TM}}{r_{TE}}\right|$ is defined as the absolute amplitude ratio between the TM and TE reflected components and $\cos\left(\Delta \right)=\cos\left(\phi_{TM}-\phi_{TE}\right)$.

Assuming the intensity of incident light to be $I_{inc}=2I_{input}$, we obtained an expression for the output light $I_{output}$ using Jones calculus given by

(2) $$I_{output}=I_{input\;}\left(0.5+0.5\tan^{2}\psi +0.5\left(\tan^{2}\psi -1\right)\cos2A+\tan\psi \cos\Delta \sin2A\right)$$

here, A is the orientation of the analyzer with respect to the TM polarization. In the proposed method, the polarization camera acts as an analyzer. Therefore, we have four different orientations of the analyzer (A), 0°, 45°, 90°, and 135° respectively.

In Equation (2), we have three unknown variables, $I_{input}$, which is half the incident intensity, tanψ, and cosΔ. Therefore, to extract the values of unknown variables, we need at least three equations. We obtain three equations by using three different analyzer orientations (A=0°,45°,135°) of the polarization camera pixels given by

(3) $$I_{0}=I_{input}\tan^{2}\psi$$

(4) $$I_{45}=I_{input}\left(0.5+0.5\;\tan^{2}\psi +\tan\psi \cos\Delta \right)$$

(5) $$I_{135}=I_{input}\left(0.5+0.5\;\tan^{2}\psi -\tan\psi \cos\Delta \right)$$

Solving the equations, the following ellipso-polarimetric parameters are obtained,

(6) $$\tan\psi =\sqrt{\frac{I_{0}}{I_{input}}},\;\;\cos\Delta =\frac{1}{2\tan\psi }\frac{I_{45}-I_{135}}{I_{input}}$$

where, $I_{input}=I_{45}+I_{135}-I_{0}$.

### 2.2. Experimental Setup

**Figure 1:**
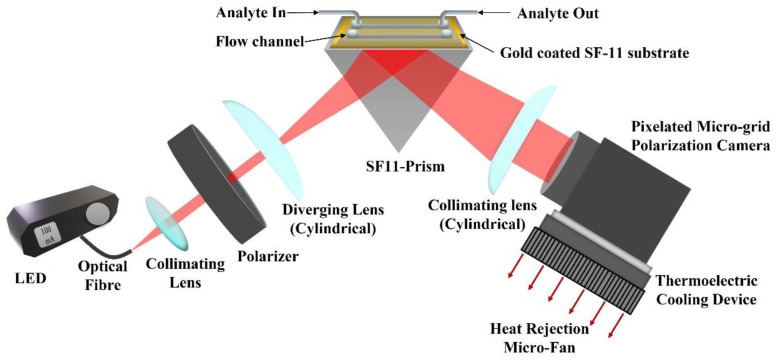
Experimental setup of the proposed method (red arrows represent heat dissipation direction using micro-fan).

The experimental setup of the proposed method is depicted in Figure 1. A 660 nm red LED (M660F1, Thorlabs Inc., Newton, NJ, USA) is used as an input light source that is operated using a constant current LED driver (DC2100, Thorlabs Inc., Newton, NJ, USA). The output light from the LED is coupled to an optical fiber (M53L02, Thorlabs Inc., Newton, NJ, USA) of 0.5 NA and 600 μm diameter. The light from the fiber is collimated using a collimation package and then passes through a linear crystal polarizer (extinction ratio of 10,000:1) oriented at a 45° angle with respect to the TM polarization direction. Therefore, light falling on the sample chip is linearly polarized with equal components of TM and TE polarization. The linearly polarized light passes through a cylindrical lens which introduces cylindrical divergence perpendicular to the propagation direction of the light (in the incidence plane). The cylindrical divergence of the light beam ensures a wide range of spatial frequencies or incident angles. The diverging beam of light hits the SPR chip, passing through a prism made of SF11 glass with a refractive index of 1.7783 [47]. The chip is made of SF11 glass coated with a thin metal film of 2 nm of titanium and 50 nm of gold, respectively. The thin metal films were deposited using the E-beam deposition technique. The chip is placed on top of the prism using a refractive index matching oil (Cargille Labs, Cedar Grove, NJ, USA) to close the gap between the chip and the prism. For calibration and finding the pixel size in refractive index units (RIU), de-ionized (DI) water and multiple concentrations of DI water with glycerol were used. The mass concentration of DI water and glycerol were 2.5%, 5%, 10%, 15%, 20%, and 25%, with corresponding refractive indices 1.3367, 1.3410, 1.3455, 1.3526, 1.3593, and 1.3685. The refractive indices of the prepared analytes were also measured using a handheld refractometer (OPTi Digital Handheld Refractometer, Bellingham & Stanley Ltd, Royal Tunbridge Wells, UK) and the two channels Photonicsys H5 SPR system. The light reflected from the chip passes through the prism and is collimated using another cylindrical lens. The collimated light is analyzed using the pixelated microgrid polarization camera (Phoenix 5MP, Lucid Vision Labs Inc., Richmond, Canada). A cooling fan was found to stabilize the signal better, so for future applications, a thermo-electric cooling (TEC) element may be attached to the polarization camera to minimize the effects of temperature-related noises.

**Figure 2:**
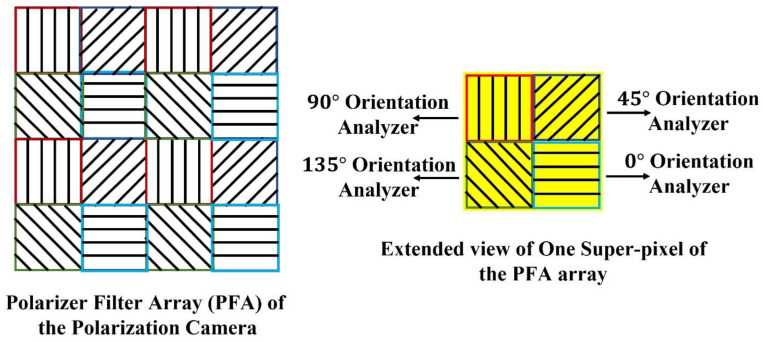
Polarization Filter Array of the Polarization camera.

The pixelated micro-grid polarization camera is sensitive to the polarization property of light. The camera sensor is based on Sony Polarlens IMX250MZR (mono) camera sensor. This polarization camera sensor consists of a polarization filter array (PFA) of microgrid wire polarizers which is placed in front of a CMOS image sensor. The PFA has on-chip micro-lenses. The PFA is placed on-chip with an air-gap nano wire-grid structure, coated with an anti-reflection material that suppresses flaring and ghosting. This on-chip placement reduces polarization crosstalk and improves the extinction ratios of the polarizers. Each super-pixel of the PFA consists of full pixels where each pixel corresponds to different micro-polarizers oriented at four different angles, 0°, 45°, 90°, and 135° respectively, which act as analyzers for the incoming linearly polarized light. The PFA and extended view of one super-pixel of the array are shown in Figure 2.

The extinction ratio of the micro-polarizers is defined as the ratio of the maximum to the minimum signal detected by a particular polarization pixel channel for a linearly polarized input. The extinction ratio is a function of the wavelength of light. The micro-grid polarizers of the sensor have an extinction ratio of ~150:1–135:1 at 633–660 nm wavelength, respectively [48,49]. Although this extinction of the analyzers is not as high as that of the crystal polarizer (10,000:1), it will have little effect on the sensor performance because it affects the absolute value of the refractive index but not its precision. The refractive index absolute value is determined by the calibration process anyway, while for the sensor, what is important is the precision. In a single shot acquisition of the polarization camera, four images are obtained, which correspond to the intensity distribution of light analyzed from four different analyzers of each super-pixel. The ellipsometric parameters of light are extracted from three of these acquired images. Therefore, the camera allows real-time monitoring of reflected SPR signal phase and intensity in a single acquisition.

### 2.3. BSA Binding Assay-Immunosensing

The SPR chips made of 50 nm gold on SF11 glass were fabricated in the nanofabrication facility of the Ben Gurion University of the Negev. From Sigma Israel, 11-mercapto-undecanoic acid (11-MUDA), Phosphate buffered saline (PBS), N-hydroxysuccinimide (NHS), 1-ethyl-3-(3-dimethylaminopropyl) carbodiimide (EDC), sodium acetate (SA), acetic acid, ethanolamine (EA), and Bovine Serum Albumin (BSA) were purchased. Absolute ethanol was procured from Romical Israel. Double-distilled water produced by a Millipore Direct-Q 3 UV (Millipore Sigma, Rehovot, Israel) was used to prepare all the buffers. Anti-BSA antibody produced in rabbits was procured from Sigma Israel. In this study, a binding assay of BSA–anti-BSA-antibody was performed to demonstrate the sensor’s performance. Briefly, medium-chain alkane thiol was used to prepare a self-assembled monolayer (SAM) on the gold substrate. For this, a cleaned gold substrate was incubated in 1% *w*/*w* 11-MUDA in absolute ethanol at 4 °C overnight. The substrate was rinsed with excess ethanol, followed by Milli-Q water, and dried with Nitrogen. After mounting the substrate on the prism, the flow channels were assembled to handle fluids. The system was equilibrated with PBS for nearly 10 min under a steady flow condition. To immobilize anti-BSA-antibody, the 11-MUDA-coated gold substrate was activated with a 10 min injection of a freshly prepared EDC/NHS solution (0.8 M EDC, 0.2 M NHS) in a 1:1 ratio for amine coupling with antibody. Before the injection of the antibody, NHS-ester was stabilized using sodium acetate buffer (10 mM, pH 4.5) for 2 min. After the immobilization of the antibody (20 μg/mL in PBS), the remaining binding sites were blocked with a 10 min injection of 1 M ethanolamine. The baseline with PBS was achieved before starting the BSA-binding assay. An association and dissociation time of 12 min and 4 min, respectively, were kept constant for the cumulative binding assay in the concentration range of 6–22 μg/mL. Further, binding kinetics studies were conducted using an excel solver (1) and Origin Pro 8.5. The details of which can be found elsewhere [50].

## 3. Results and Discussion

### 3.1. Refractive Index Measurements and Calibration

Refractive index measurements for the different concentrations of de-ionized water, ethanol and glycerol solutions as analytes were performed on the proposed experimental setup for calibration and verification purposes. Six different concentrations of de-ionized water and glycerol were used for the experiment. During the measurements, the input polarizer remained fixed at an angle of 45° with respect to the TM polarization of the light. The LED current was set to 100 mA. The exposure time of the polarization camera was set to 4000 μs with 24-bit intensity resolution, 5.278 Hz acquisition frame rate, and 2448×2048 pixels image size. All these parameters remained constant throughout the measurements.

**Figure 3:**
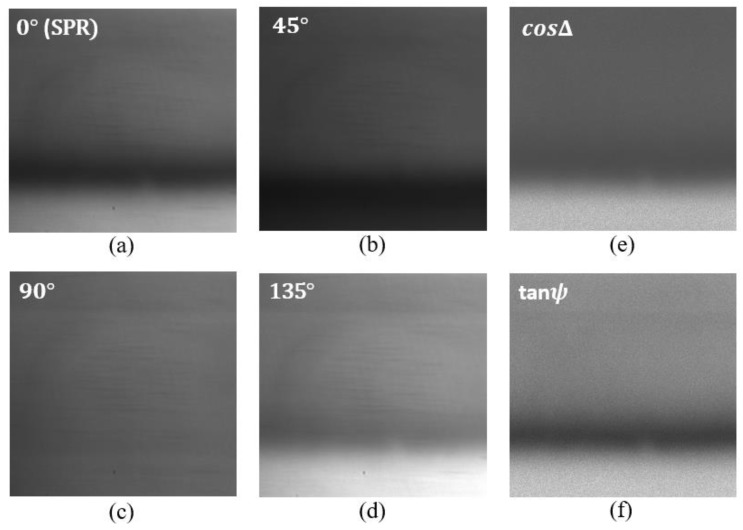
Intensity distribution of the reflected beam from the SPR chip corresponding to di-ionized water for different orientations of the polarization camera micro-grid polarizer. (**a**) 0° orientation, (**b**) 45° orientation, (**c**) 90° orientation, and (**d**) 135° orientation. (**e**,**f**) corresponds to the intensity distribution of cosΔ and tanψ, respectively.

For each analyte, four intensity images were grabbed corresponding to four different orientations of the polarization camera 0°, 45°, 90° and 135°. Measurement results for the de-ionized water as analyte are shown in Figure 3 below. Figure 3a represents the SPR intensity distribution and corresponds to the image $\left(I_{0}\right)$ for 0° orientation of the polarization camera analyzer. In this case, the analyzer only allows passing the TM polarization component of light. Figure 3b corresponds to the image $\left(I_{45}\right)$ for 45° orientation of the analyzer. In this case, the transmission axes of the input polarizer and the analyzer remain perpendicular to each other. Figure 3c corresponds to the image $\left(I_{90}\right)$ for 90° orientation of the analyzer. In this case, the analyzer only allows the TE component of light to pass; therefore, we do not observe any SPR signature in this image. Figure 3d corresponds to the image $\left(I_{135}\right)$ for 135° orientation of the analyzer. In this case, the transmission axes of the input polarizer and the analyzer remain parallel to each other. Each pixel in the obtained images corresponds to the intensity measurements for one combination of polarizer and analyzer orientations. The obtained images are used to compute the polarimetric parameters tanψ and cosΔ using Equation (6). For each pixel in the images $I_{0}$, $I_{45}$, and $I_{135}$ the corresponding tanψ and cosΔ images were found. The calculated images of tanψ and cosΔ are shown in Figure 3f and Figure 3e, respectively.

**Figure 4:**
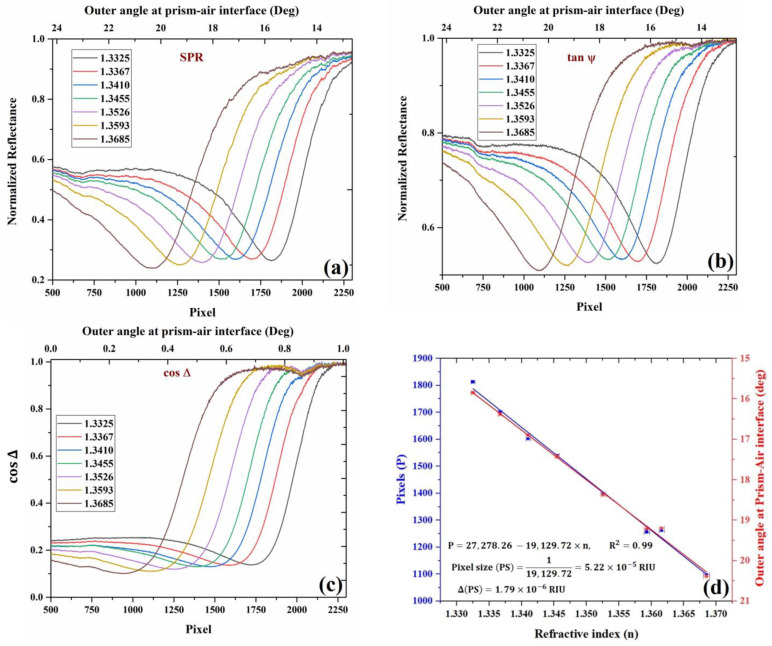
Experimental curves for (**a**) SPR, (**b**) tanψ, (**c**) cosΔ, and the variation of SPR dips with different refractive index analytes. The curves were obtained using different concentrations of water–glycerol solution and de-ionized water. For each analyte, the curves shown above are calculated from four intensity images acquired from polarization camera, which corresponds to four different orientations of the analyzer, and (**d**) Change in the position of SPR with a change in the RI of the analyte in pixels (blue) and outer angle (red) in degrees. Ethanol RI point is also added to the figure.

Furthermore, for analysis, the image profiles of the obtained tanψ and cosΔ images are plotted for all the analytes as a camera signal versus the pixel number. The variation in the pixel number is translated into angular shift or refractive index variation using a calibration curve. For each analyte, the change in pixel position is translated into the change in the outer angle of incidence in the air–prism interface. For this calibration, a theoretical simulation of angular SPR in KR configuration was performed using the 2×2 Abele’s matrix approach and the Drude–Lorentz dispersion model for the metals. Image profiles averaged along the columns of the obtained images are shown in Figure 4 below, along with the pixel-angle calibration curve. Note that as the pixel number increases, the angle decreases because the image is inverted. As a sensor, this inverted presentation does not matter much as one is interested in the shift of the pixel/angle and not the absolute value or whether it decreases or increases with the refractive index.

All the required image-processing tasks were performed using the image-processing toolbox of MATLAB. Figure 4a corresponds to the image profile of the $I_{0}$ image, which represents the SPR intensity distribution. The intensity profiles of the SPR image for each analyte were calculated and plotted as a function of pixels. The intensity profile was calculated by summing over the columns of the image, therefore taking the mean value of pixels in each row in the image. The corresponding change in the outer incidence angle on the air–prism interface with pixels is shown in the figure. The position of the SPR dips represents normalized reflectance dip changes with respect to changes in the refractive index of the analyte. Figure 4b corresponds to the image profiles of tanψ as a function of pixels and the corresponding change in the outer angle (upper axis) at the air–prism interface for different analytes. Figure 4c corresponds to the image profiles of cosΔ for different analytes. In the figure, the horizontal axis represents the number of pixels representing the location of cosΔ phase jump in the intensity image, and the vertical axis represents the cosΔ which corresponds to the phase difference between the TM and TE components of light. It can be observed that there is a phase jump at SPR, which shifts to a new location upon a change in the analyte refractive index.

The calibration data are presented in Figure 4d, where the shift in SPR dip position in pixels is plotted versus the RI of the analyte showing linear relation. From the slope, we can easily find the pixel size to be $PS=1/19,307=5.18\times 10^{-5}\;\mathrm{RIU}$. The error is the slope is estimated to be $\Delta \left(PS\right)=1.7\times 10^{-6}\;\mathrm{RIU}$, which is found from linear regression with the experimental errors in the pixel position and the RI. The error in the pixel position is 0.2 pixels, while the RI measurement error based on the Abbe refractometer is 0.000319 based on the company manual. After the calibration, we checked again the values obtained for DI water and ethanol, and other concentrations of glycerol, which were found to be agreed with the known values to within the pixel size.

### 3.2. Advantage of Real-Time Ellipsometric SPR Measurement with Fluctuating Light Source Environment

Ellipsometry has the advantage of being a self-referenced measurement, and therefore it compensates for fluctuations in the light source intensity. This is important when lasers and thermal light sources are used. In this sense, it is superior to interferometry, and it can be considered a common path orthogonal polarization interferometry. The interference of the two orthogonal components occurs at the analyzer plane for the components of the two beam parallel to the polarizer axis. In this subsection, we demonstrate this fact using a laser diode that has high fluctuations with time and with LED, which is about an order of magnitude less noisy.

**Figure 5:**
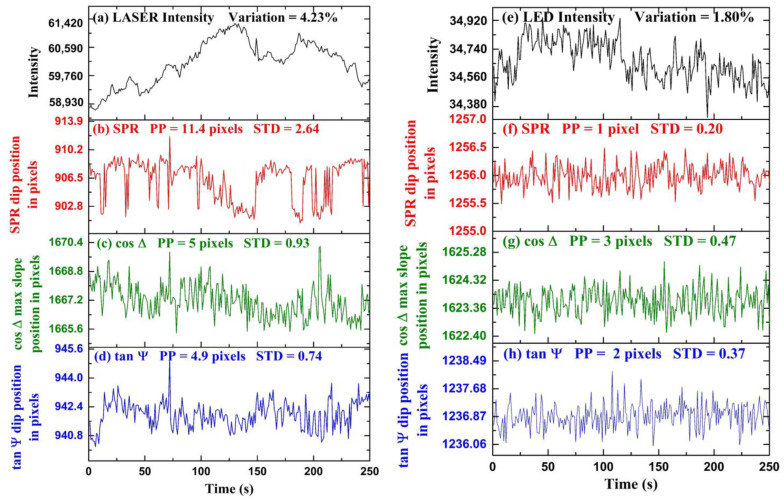
Temporal fluctuations of different optical signals when LASER is used: (**a**) Intensity fluctuations of LASER diode, (**b**) SPR dip position, (**c**) cosΔ maximum slope position, (**d**) tanψ dip position. Similarly, for LED: (**e**) Intensity fluctuations of LED, (**f**) SPR dip position, (**g**) cosΔ maximum slope position, and (**h**) tanψ dip position.

Experimental results for time-series measurements are shown in Figure 5. In the first part of the experiment, we demonstrated the stability of polarimetric parameters cosΔ and tanψ over the conventional SPR in a fluctuating light source environment. The tanψ fluctuations are obtained by tracking the position of the dip of tanψ with time. The cosΔ fluctuations are obtained by detecting the position of the maximum slope of cosΔ profile with time. In Figure 5a, intensity fluctuations of a laser diode (CPS650F 633 nm, Thorlabs Inc., Newton, NJ, USA) measured over time are shown. The intensity fluctuations were measured using a spectrometer (AvaSpec-2048, Avantes, Apeldoorn, The Netherlands). The maximum intensity variation of the laser light is calculated to be 4.23% from the average intensity value throughout the period. We performed a time-series measurement using de-ionized water as an analyte and fluctuating laser diode as an input light source. The polarization camera frame rate was chosen to be 1 F/s, the exposure time was set to 3000 μs, and a 5 min video was captured during the measurement. The video was captured in the combined acquisition of four different orientation images in a single frame. Each frame in the video is a grid consisting of four images $I_{0}$, $I_{45}$, $I_{90}$, and $I_{135}$ respectively, and tanψ and cosΔ were calculated using Equation (6). We extracted the frames of video and acquired the four images separately for further computation using the MATLAB image processing toolbox. Figure 5b represents the SPR dip position as a function of pixels with time. The standard deviation and peak-to-peak change of the fluctuations in the position of SPR in the form of pixels are calculated to be 2.64 pixels and 11.4 pixels, respectively. Figure 5c represents the variation in the maximum change position of cosΔ with time. The horizontal axis represents time, and the vertical axis represents the position of the maximum slope of cosΔ in pixels. The standard deviation and peak-to-peak change of the fluctuations in the position of cosΔ in units of pixels are calculated to be 0.93 pixels and 5 pixels, respectively. Figure 5d represents the tanψ dip position variation with time. The horizontal axis represents time, and the vertical axis represents the position of tanψ dip in pixels. The standard deviation and peak-to-peak variation of the fluctuations in the position of tanψ in the form of pixels are calculated to be 0.74 pixels and 4.9 pixels, respectively. It can be observed that because ellipsometry is a self-referenced measurement as compared to conventional SPR, the precision is better. It has been found that ellipsometric parameters tanψ and cosΔ have fewer fluctuations as compared to SPR; therefore, it provides better stability as compared to SPR in fluctuating intensity light source environments. The cooling of the camera was found to help significantly, and we believe that further improvements are possible with better-controlled cooling to lower temperatures.

In the second part of the experiment, we performed time-series measurements of tanψ, cosΔ and SPR using a more stable LED light source, as shown in Figure 5. The LED current was set to 100 mA, and the camera exposure time was set to 3000 μs with a frame rate of 1 F/s. For the experiment, de-ionized water was taken as an analyte, and a 5 min video was captured using the polarization camera. Using the process discussed before, we extracted the images $I_{0}$, $I_{45}$, $I_{90}$, and $I_{135}$ and tanψ and cosΔ calculated using Equation (6). According to these results, when the input light source has very little intensity fluctuations, such as the LED, less than the precision of the ellipsometric measurement, then there is no apparent advantage of monitoring tanψ over the conventional SPR for the time-series sensing experiments. Figure 5e represents the intensity fluctuations of a LED (Thorlabs DC2100) as an input light source. The horizontal axis represents time, and the vertical axis represents intensity counts. The maximum intensity fluctuations of the LED are calculated to be 1.35% from the average intensity value throughout the measurement time. It can be observed that the LED is a more stable light source as compared to the laser diode shown in Figure 5a by nearly an order of magnitude in intensity fluctuations. In Figure 5f–h, the fluctuations in the position SPR dip, position of maximum slope in cosΔ, and the position of tanψ dip in pixels with time are shown, respectively. The horizontal axis represents time, and the vertical axis represents the position of the dip in pixels. The standard deviation and peak-to-peak variation in the fluctuations of maximum slope in cosΔ in pixels are calculated to be 0.47 and 3 pixels. The standard deviation and peak-to-peak variation of the fluctuations in the position of tanψ in the form of pixels are calculated to be 0.37 pixels and 2 pixels. The standard deviation and peak-to-peak variation of the fluctuations in the position of SPR in the form of pixels are calculated to be 0.20 pixels and 1 pixel. It is found that there is no advantage to monitoring polarimetric parameters cosΔ and tanψ over the SPR dip when using such as stable light source environment. It is possible that because the SPR measurement precision in the LED case is already in the sub-pixels regime, any further improvement in the measurement will not make it better. Reducing the pixel size further, in this case, might help and show the further advantage of real-time ellipsometry even with quiet light sources such as LEDs.

For the stable light source (LED), the stability of polarimetric parameters cosΔ and tanψ improved compared to fluctuating light source (LASER diode); however, it offers no advantage over SPR. In the case of LASER, the intensity fluctuations were large, and the polarimetric parameters were more stable than SPR because of self-referenced measurement. In the case of LED, the intensity fluctuations were small; therefore, the self-referenced advantage could no longer provide a more stable polarimetric information parameter than SPR. We should point here to the conclusion of reference [51] that states the phase measurement is not necessarily better than intensity measurement because to measure the phase, one, in fact, measures intensity. While this can be true if the phase measurement is performed with interferometry, our experiments show that this is not true if the measurement is self-referenced such as when performed with ellipsometry. Hence, we propose that real-time ellipsometry can provide the ultimate limit for SPR detection in avoiding the effect of light source fluctuation; however, other sources of noise need to be reduced independently, such as thermal noise, mechanical drifts, as well as the reduction of the pixel size of the camera.

### 3.3. Dynamical Measurements

Dynamic changes in the analyte refractive index are essential for monitoring variations in the concentration of molecules bounded to the surface with time or for analyzing the binding kinetics between molecules. Dynamical change measurements are essential, having various applications in the pharma industry. The rotating polarizer-based three-point ellipsometric SPR sensor requires the sequential acquisition of images at different angles of the polarizer. This method is limited by its real-time measurement applications as the images for different orientations of the polarizer are captured at different time instances. Our method involves the simultaneous acquisition of images corresponding to four different orientations of the polarizer using a pixelated microgrid polarization camera. The simultaneous acquisition enables the monitoring of ellipsometric parameters for real-time applications.

The effect of time delay between the sequential acquisition of images at different polarizer angles is demonstrated here, which is similar to the rotating polarizer method, and we show the advantage of the proposed method for real-time dynamical change measurements. The dynamical change of ellipsometric parameter tanψ obtained from the sequential image acquisition (similar to a rotating polarizer) is compared to the proposed method. Based on the proposed setup, we took a 5 min video using a polarization camera at 4500 μs of exposure time and a frame rate of 5 F/s. In the experiment, de-ionized water was used as the first analyte; then, we started flowing 20% water–glycerol solution as a second analyte on the SPR sensor chip.

**Figure 6:**
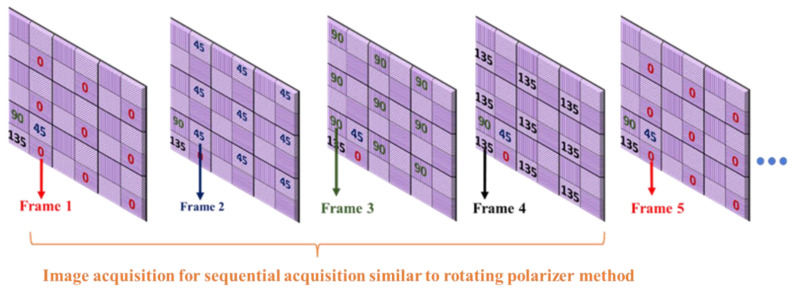
Acquiring four different polarization orientation images from a sequence of frames to demonstrate the sequential acquisition (rotating polarizer method) from a pixelated micro-grid polarization camera.

For the proposed method, tanψ was extracted using images $I_{0},I_{45},I_{90}$, and $I_{135}$, acquired from a single shot of the polarization camera using Equation (6). For the sequential acquisition method, we used the same 5 min captured video but considered only one polarizer position from each frame ($I_{0},I_{45},I_{90}$ or $I_{135}$) and neglected the other three polarizer angles, which is equivalent to the rotating polarizer method, where only one polarizer orientation image is captured in a single shot. Each frame acquired from the polarization camera comprises super-pixels, which are collections of four pixels, each representing different polarizer orientations. We extracted $I_{0}$ image from the first frame, $I_{45}$ from the second frame, $I_{90}$ from the third frame, and $I_{135}$ from the fourth frame, as shown in Figure 6. Then we calculated tanψ for the sequential acquisition method, using Equation (6) as before.

**Figure 7:**
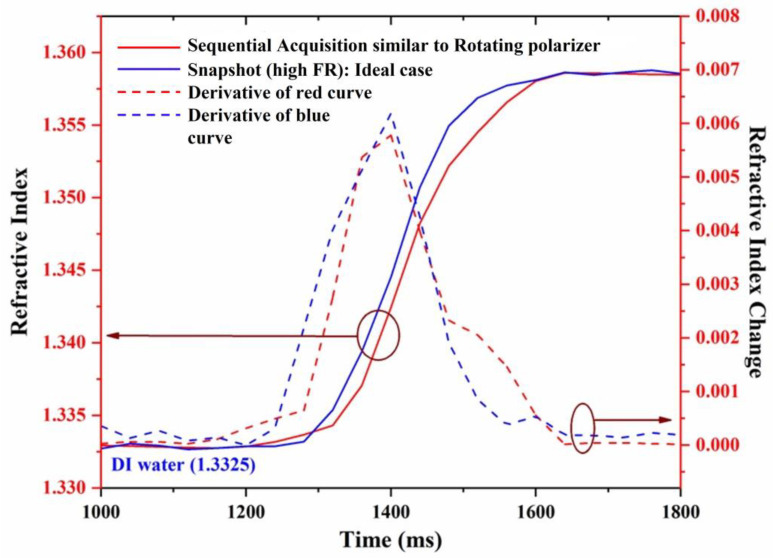
Dynamic change measurement of refractive index.

The dynamic change in the refractive index is represented by the change in the ellipsometric parameter tanψ, as shown in Figure 7. The horizontal axis represents time, the left vertical axis represents the refractive index, and the right vertical axis represents the change in the refractive index. The blue and red curves represent the dynamical change measured from the proposed method and the sequential acquisition method, respectively.

The dashed curves represent the slope of the dynamical change curves for the proposed method (blue curves) and the sequential acquisition method (red curves). It is observed from the dynamical change curves that the measurement of tanψ extracted from the proposed method presents different behavior compared to a sequential acquisition (rotating polarizer) method. Although the kinetics of the measurement obtained from the two methods look similar, the one obtained in real-time looks more regular curve, as can be more clearly seen from the slope curves (dashed).

### 3.4. BSA-Binding Assay and Kinetics

**Figure 8:**
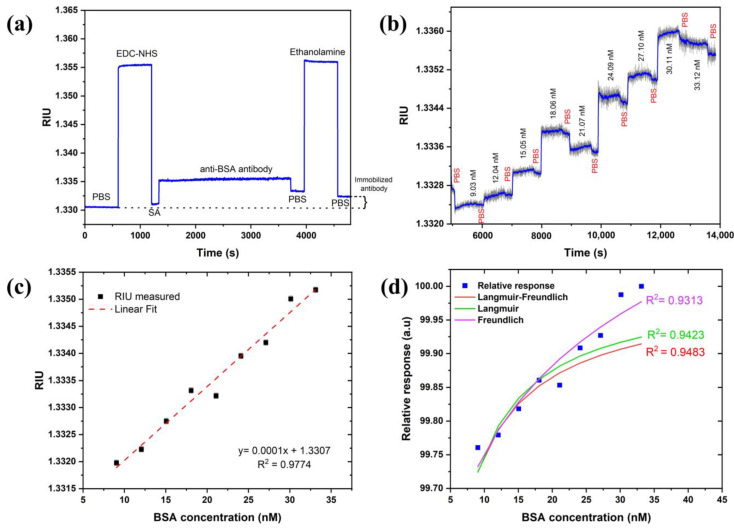
(**a**) Monitoring functionalization steps of anti-BSA antibody, (**b**) BSA-binding assay sensogram, (**c**) calibration curve of SPR response of BSA–anti-BSA antibody binding, and (**d**) adsorption isotherm fitting to relative and normalized response of experimental data.

The BSA–anti-BSA-antibody binding assay demonstrated the sensor’s potential for biosensing. Anti-BSA antibody was allowed to immobilize for 40 min (min) to obtain a better coverage on the sensor surface. Figure 8a revealed that a change of 0.0018 RIU was observed after the immobilization steps were completed, confirming the coupling of the antibody with carboxylated SAM. A sensogram of the cumulative binding assay with association and dissociation times of 12 min and 4 min, respectively, are shown in Figure 8b. The sensogram was smoothened to witness association and dissociation events upon injection of increasing BSA concentrations and PBS buffer. This further confirms the binding of BSA with immobilized antibodies.

A calibration curve was constructed from the subsequent baselines (PBS) obtained after the dissociation curves and fitted to linear regression in Figure 8c. The limit of detection (LoD) was calculated using the expression: LoD=k×STD/S where k is the confidence limit (k=3), S is the sensitivity RIU/nM defined the slope of the linear regression curve of Figure 8c, and STD in RIU is the standard deviation measured from the sensor response. The STD in pixels is estimated as 0.2–0.4 pixels based on Figure 5 without any averaging for the LED case. The pixel size is estimated as $5.18\times 10^{-5}\;\mathrm{RIU}$ so the LoD in RIU is $\left(3–6\right)\times 10^{-5}$ RIU. Therefore, the LoD in the concentration of BSA is thus 0.3–0.6 nM valid for the linear range of concentrations of 6–22 μg/mL (or 9.03–33.12 nM) of BSA. This suggests that one can obtain an even lower detection limit with the current set of parameters. It should also be mentioned that with averaging and smoothing, one should be able to reduce the LoD both in the RIU and the concentration by another order of magnitude. We checked that the STD reduces to 0.02 by averaging 50 times which did not affect the speed using the moving average approach. The investigation of the dissociation constant ($K_{D})$ was also performed. For this, adsorption isotherms (Langmuir, Freundlich, and Langmuir–Freundlich) were used and fitted to the relative response of experimental data, as shown in Figure 8d. Langmuir and Langmuir–Freundlich isotherms were fitted using an excel solver from Umpleby et al. [52], whereas the Freundlich isotherm was fitted using the alometric1 (classical Freundlich) function from Origin pro 8.5 [50]. All the fitting parameters were calculated from the method mentioned in the above reference to compare and find out the best-fitted model (Table 1). As expected, the Langmuir–Freundlich model fitted the best since an antibody (with two binding sites)–antigen pair usually shows a homogenous but cooperative binding. The dissociation constant was obtained to be 0.012 nM, which lies within the acceptable range of a typical antibody–antigen pair measured using SPR in other studies [53].

## 4. Summary

In this work, a real-time parallel ellipsometric SPR setup is proposed operating in the angular KR configuration using a diverging beam and polarization camera. The ellipsometric parameters tanψ and cosΔ were extracted using images of the three different polarizations of the pixelated microgrid polarization camera. BSA–anti-BSA-antibody binding assay and kinetics experiment demonstrated the potential of the approach for biosensing, showing a detection limit in the 0.1 nM range or 10^−5^ in RIU, while using averaging and smoothing of the data, improved it by an order of magnitude. Therefore, one can conclude that the achievable LOD is 10 pM of BSA or 1RU (10^−6^). Of course, better thermal stabilization and a camera with a larger number of pixels can bring the LOD down by another order of magnitude (1 pM). It should be noted that in many estimates of the LOD, the different works [1,2] ignore drifts that occur at the time scale of minutes, which causes the LOD to be reported as less than the reported values usually. In our setup, as can be seen, the measurements are performed for a period of 5–20 min. The setup can be used for real-time phase SPR sensing applications exhibiting an advantage as compared to previous works on polarimetric SPR sensors. The stability of ellipsometric parameters cosΔ and tanψ were compared to SPR in different light source environments showing that the ellipsometric parameters are superior to standard SPR dip position measurement with a fluctuating light source such as a laser diode. Using a quieter LED source, the improvement of ellipsometric parameters was not noticeable, possibly because the system had already approached its detection limit of sub-pixel resolution. If the pixel size is reduced further, as well as other noise sources such as thermal and mechanical, we expect the ellipsometric measurement to show improvement even with LED as a light source. Hence, following this work, we propose that real-time ellipsometry, as a self-referenced approach, can provide the ultimate resolution limit for SPR systems.

## Figures and Tables

**Table 1:** Fitting parameters for binding isotherms.

Fitting Parameter	Langmuir	Freundlich	Langmuir–Freundlich
N_t_	100	−	100
a	40	99.31	50
m	1	0.002	0.9
R^2^	0.9423	0.9313	0.9483
K_D_	0.025 nM	−	0.012 nM

## Data Availability

Data is unavailable due to privacy or ethical restrictions.
